# Missing Traffic Data Imputation with a Linear Generative Model Based on Probabilistic Principal Component Analysis

**DOI:** 10.3390/s23010204

**Published:** 2022-12-25

**Authors:** Liping Huang, Zhenghuan Li, Ruikang Luo, Rong Su

**Affiliations:** School of Electrical and Electronic Engineering, Nanyang Technological University, Singapore 639798, Singapore

**Keywords:** missing data, urban traffic sensing, probabilistic, principal component analysis

## Abstract

Even with the ubiquitous sensing data in intelligent transportation systems, such as the mobile sensing of vehicle trajectories, traffic estimation is still faced with the data missing problem due to the detector faults or limited number of probe vehicles as mobile sensors. Such data missing issue poses an obstacle for many further explorations, e.g., the link-based traffic status modeling. Although many studies have focused on tackling this kind of problem, existing studies mainly focus on the situation in which data are missing at random and ignore the distinction between links of missing data. In the practical scenario, traffic speed data are always missing not at random (MNAR). The distinction for recovering missing data on different links has not been studied yet. In this paper, we propose a general linear model based on probabilistic principal component analysis (PPCA) for solving MNAR traffic speed data imputation. Furthermore, we propose a metric, i.e., Pearson score (p-score), for distinguishing links and investigate how the model performs on links with different p-score values. Experimental results show that the new model outperforms the typically used PPCA model, and missing data on links with higher p-score values can be better recovered.

## 1. Introduction

Traffic data generated by loop detectors or floating cars in urban road networks serve as the foundation for various data-driven applications in intelligent transportation systems, including traffic forecasting and traffic control [1,2,3]. However, even with ubiquitous sensing data, the missing data problem is almost inevitable due to either detector faults or a limited number of probe vehicles operating as mobile sensors in road networks, which means not each road in the network is covered by a detector or traveled by a probe vehicle in each minute [4,5,6]. Such an issue of missing traffic data poses obstacles for many further data-driven explorations in both academic and industrial fields, e.g., the link-based traffic status modeling, and network-wise traffic dynamics capturing [7,8]. Hence, accurate and reliable imputation is a basic need for such kind of incomplete data for the downstream explorations.

Many efforts have been made for estimating the missing traffic data on multiple traffic datasets, resulting the generative probabilistic model [9], the matrix decomposition and tensor factorization models [10,11,12], the autoencoder model [13], the fusion models [14]. Some basic mathematical models are also adopted, including the autoregressive integrated moving average (ARIMA) model, the Bayesian networks (BNs) method, the Markov chain Monte Carlo (MCMC) method, and the K-nearest neighbors (KNN) model, which are all tested in [15] for traffic missing data imputation.

The studies in [16] have validated that the matrix decomposition-based method is not capable for recovering missing data when the missing percentage large. The tensor models are based on the global structure capturing, and it is faced with challenging to permutation in the spatial and temporal dimension [17]. The probabilistic principal component analysis (PPCA) model [18] also plays a major role in missing data completion due to its generative feature [19]. Observations are assumed to be generated from a low dimensional space, with which the missing data can be recovered by optimizing the generative parameters using the observations [20].

Although many studies have focused on tackling this kind of problem, existing studies focus on the situation that the data are missing at random. Specifically, missing data can be classified into missing at random, missing completely at random, and missing not at random (MNAR) [16]. MNAR data always exist in the practical scenarios, and it is more challenging to estimate the missing values, which is the target of this paper.

The studies in [15] demonstrate that the PPCA model yields best performance among ARIMA, BNs, MCMC, and the KNN models, and in the research in [21], it has been certified that the PPCA model outperforms the basic tensor decomposition method. Hence, in this study, we set the PPCA model as a basis and further improve the PPCA model for tackling the MNAR traffic data. Additionally, the missing data on different links or sensors may be of different levels of challenges for data completion. Hence, there is also a need to distinguish different scenarios that missing data are on different links or sensors. Instead of the centrality of a sensor in the network, we utilize the time series correlations to define a metric for distinguishing the role of a link in the traffic network. Such a metric is adaptive to the scenario that sensors or links are anonymous. Contributions of this work are summarized as below:

- We design a metric, p-score to denote the relative importance of links in terms of time series observations, which is used to distinguish the links with missing values.
- We propose a linear model for the MNAR traffic data imputation, which is based on the probabilistic principal component analysis.
- We conduct experiments on a real-world traffic dataset using the model and the proposed metric. Experimental results show missing data on links with higher p-score values can be better recovered. Moreover, testing on the real-world dataset, the results of the proposed model on links with the lowest p-score value also outperforms the typically used PPCA model.

The remainder of the paper is structured as follows. Section 2 presents the problem statement of the missing traffic data imputation. Section 3 the details of the proposed model. Section 4 shows the outcome of the experimental evaluation results, Section 5 presents a short discussion of the potential application scenarios of the proposed method, and finally, Section 6 gives the conclusions of this paper and the directions for future studies.

## 2. Problem Statement

Let $Y\in {\mathbb{R}}^{n\times p}$ be a traffic data organization matrix with each element $Y_{ij}$ denoting the $i^{}$ observation of a link j.

We assume that the traffic data are missing and links with missing values are organized as $Y_{\cdot m_{1}},\;Y_{\cdot m_{2}},\ldots ,Y_{\cdot m_{d}}$, which is indexed by $ℳ:=\left\{m_{1},m_{2},\ldots ,m_{d}\right\}\subset \left\{1,\ldots ,p\right\}$ with d<p are supposed to have missing values. Here, ℳ is the link set that has missing values. Other values in Y are observed.

We label the missing status of $Y_{ij}$ with another variable, written as

(1) $$\Omega_{ij}=\{\begin{array}{c} 0,\;\;Y_{ij}\;is\;missing\;\\ 1,\;otherwise\; \end{array}$$

Traffic sensing in urban road networks is faced with the missing data, or data sparsity problem. We construct the traffic matrix Y with all missing values in columns ℳ. The missing data imputation problem is to estimate these missing values, i.e., ${\hat{Y}}_{im},\;m\in ℳ$, where $\Omega_{im}=0$.

## 3. Methodology

### 3.1. PPCA

Assuming that the target variable is organized as a matrix *Y*, and it can be drawn from X of a low rank by linear combination, written as

(2) Y=1α+XA+ϵ

here, $Y\in {\mathbb{R}}^{n\times p}$, where n is the sample number and p is the number of variables in the determination system. Specifically, in our link-based missing data imputation problem, p is the total number of links.

$X={\left(X_{1\cdot }|\cdots |X_{n\cdot }\right)}^{T}$ is the latent variable. $X\in {\mathbb{R}}^{n\times r}$, and the row is drawn from a Gaussian distribution with zero mean, i.e., $X_{i\cdot }\sim N\left(0_{r},Id_{r\times r}\right)$. Here, r<min{n,p}, indicating a lower dimension. A is the loading matrix of rank $r,\;A\in {\mathbb{R}}^{r\times p}$. $\epsilon ={\left(\epsilon_{1\cdot }|\cdots |\epsilon_{n\cdot }\right)}^{T}$ is a model error, and each row $\epsilon_{i\cdot }\sim N\left(0_{p},\sigma^{2}Id_{p\times p}\right)\in {\mathbb{R}}^{p}$, which also has a zero mean. $1={\left(1,\ldots ,1\right)}^{T}\in {\mathbb{R}}^{n}$, $\alpha =\left(\alpha_{1},\cdots ,\alpha_{p}\right)\in {\mathbb{R}}^{p}$. Given the linear expression above, the mean value of each sample of Y is α.

### 3.2. Missing Variables Differentiation Based on Time Series

Assume that we have missing data on two different variables, $Y_{\cdot j},Y_{\cdot k}$, with the same percent, the imputation accuracy can be different due to the variable’s role in the whole variable set. In the traffic missing data imputation problem, two links, $Y_{\cdot j},Y_{\cdot k}$, may have different correlations to other links. In this section, we prose a metric to differentiate the role of each link.

The observation of each link is also a time series. We first adopt the Pearson correlation coefficient to estimate the correlation between each pair of time series, which is calculated as

(3) $$\rho \left(Y_{\cdot j},Y_{\cdot k}\right)=\frac{Cov\left(Y_{\cdot j},Y_{\cdot k}\right)}{\sigma_{Y_{\cdot j}}\sigma_{Y_{\cdot k}}}$$

By calculate the Pearson correlation among each pair of variables, we can obtain a correlation matrix, written as

(4) $$P=\left[\begin{array}{ccc} 1 & \cdots & \rho \left(Y_{\cdot 1},Y_{\cdot n}\right)\\ \vdots & \ddots & \vdots \\ \rho \left(Y_{\cdot n},Y_{\cdot 1}\right) & \cdots & 1 \end{array}\right]$$

We define a Pearson score (p-score) for each variable to differentiate the variables in ℳ, which is calculated as

(5) $$P_{score}\left(Y_{\cdot j}\right)=\sum_{k\in \left\{1,\ldots ,n\right\}}P_{jk}$$

The variable $Y_{\cdot j}$ that obtains a higher p-score value than $Y_{\cdot k}$ denotes it has higher correlation to other links. Such a metric can differentiate the variables in terms of the time series observations. When $P_{score}\left(Y_{\cdot j}\right)>P_{score}\left(Y_{\cdot k}\right)$, and the two links have the same missing data percentage, the imputation accuracy for $Y_{\cdot j}$ should be higher than that of $Y_{\cdot k}$.

### 3.3. Preliminaries and Assumptions

Assume that we have missing data on two different variables, $Y_{\cdot j},Y_{\cdot k}$ with the same percent, the imputation accuracy can be different due to the variable’s role in the whole variable set. In the traffic missing data imputation problem, two links $Y_{\cdot j},Y_{\cdot k}$ may have different correlations to other links. In this section, we prose a metric to differentiate the role of each link.

Assume that we have a D dimensional Gaussian distribution, written as

(6) $$N\left(x|u,\Sigma \right)=\frac{1}{{\left(2\pi \right)}^{D/2}}\frac{1}{{\left|\Sigma \right|}^{1/2}}\exp\left(-\frac{1}{2}{\left(x-u\right)}^{T}\Sigma^{-1}\left(x-u\right)\right)$$

where u is a *D*-dimensional mean vector, Σ is a D×D covariance matrix, |Σ| denotes the determinant of Σ. Then, we partition the *D*-dimensional vector into two parts, written as

(7) $$x=\left(\begin{array}{c} x_{a}\\ x_{b} \end{array}\right)$$

Correspondingly, the mean vector and the covariance matrix are, respectively partitioned into two parts and four parts, written as below.

(8) $$u=\left(\begin{array}{c} u_{a}\\ u_{b} \end{array}\right),\;\Sigma =\left(\begin{array}{cc} \Sigma_{aa} & \Sigma_{ab}\\ \Sigma_{ba} & \Sigma_{bb} \end{array}\right)$$

We further utilize another variable Λ to denote the inverse matrix of the covariance matrix, defined as

(9) $$\Lambda \equiv \Sigma^{-1}=\left(\begin{array}{cc} \Lambda_{aa} & \Lambda_{ab}\\ \Lambda_{ba} & \Lambda_{bb} \end{array}\right)$$

Note that, we have the theory of matrix inverse as

(10) $${\left(\begin{array}{cc} A & B\\ C & D \end{array}\right)}^{-1}=\left(\begin{array}{cc} M & -MBD^{-1}\\ -D^{-1}CM & D^{-1}CMBD^{-1} \end{array}\right)$$

(11) $$M={\left(A-BD^{-1}C\right)}^{-1}$$

Hence, for the inverse of the covariance matrix, we have

(12) $${\left(\begin{array}{cc} \Sigma_{aa} & \Sigma_{ab}\\ \Sigma_{ba} & \Sigma_{bb} \end{array}\right)}^{-1}=\left(\begin{array}{cc} \Lambda_{aa} & \Lambda_{ab}\\ \Lambda_{ba} & \Lambda_{bb} \end{array}\right)$$

where we care about the expression of $\Lambda_{aa}$ and $\Lambda_{ab}$, written as

(13) $$\Lambda_{aa}={\left(\Sigma_{aa}-\Sigma_{ab}\Sigma_{bb}{}^{-1}\Sigma_{ba}\right)}^{-1}$$

(14) $$\Lambda_{ab}=-{\left(\Sigma_{aa}-\Sigma_{ab}\Sigma_{bb}{}^{-1}\Sigma_{ba}\right)}^{-1}\Sigma_{ab}\Sigma_{bb}{}^{-1}$$

For the Gaussian distribution, the exponent parts can be expanded as

(15) $$-\frac{1}{2}{\left(x-u\right)}^{T}\Sigma^{-1}\left(x-u\right)=-\frac{1}{2}x^{T}\Sigma^{-1}x+x^{T}\Sigma^{-1}u+const$$

when we partition the D-dimensional vector into two parts $x={\left(x_{a},x_{b}\right)}^{T}$, then the exponent part of the Gaussian distribution can be expanded into

(16) $$-\frac{1}{2}{\left(x-u\right)}^{T}\Sigma^{-1}\left(x-u\right)=-\frac{1}{2}{\left(x_{a}-u_{a}\right)}^{T}\Lambda_{aa}\left(x_{a}-u_{a}\right)-\frac{1}{2}{\left(x_{a}-u_{a}\right)}^{T}\Lambda_{ab}\left(x_{b}-u_{b}\right)-\frac{1}{2}{\left(x_{b}-u_{b}\right)}^{T}\Lambda_{ba}\left(x_{a}-u_{a}\right)-\frac{1}{2}{\left(x_{b}-u_{b}\right)}^{T}\Lambda_{bb}\left(x_{b}-u_{b}\right)\;$$

We assume that $x_{b}$ is known in advance, so it can be regarded as a constant. Hence, the first order of $x_{a}$ is written as

(17) $$x_{a}{}^{T}\left\{\Lambda_{aa}u_{a}-\Lambda_{ab}\left(x_{b}-u_{b}\right)\right\}$$

which should have the same expression of the original expression for the first order part written as $x^{T}\Sigma^{-1}u$. For $x^{T}\Sigma^{-1}u$, when we consider the $x_{b}$ is known, then $\Sigma^{-1}u$ can be written as $\Sigma_{a|b}{}^{-1}u_{a|b}$, which should be equal to $\Lambda_{aa}u_{a}-\Lambda_{ab}\left(x_{b}-u_{b}\right)$, written as

(18) $$\Lambda_{aa}u_{a}-\Lambda_{ab}\left(x_{b}-u_{b}\right)=\Sigma_{a|b}{}^{-1}u_{a|b}\;$$

Hence, we have the expression the estimated value of $u_{a|b}$ written as conditional Gaussian distribution

(19) $$u_{a|b}=\Sigma_{a|b}\left\{\Lambda_{aa}u_{a}-\Lambda_{ab}\left(x_{b}-u_{b}\right)\right\}$$

where $\Lambda_{aa}$ and $\Lambda_{ab}$ are already known as above.

Based on the conditional Gaussian distribution, we replace the $x_{b}$ part with $\left({\left(Y_{\cdot k}\right)}_{k\in \bar{ℳ}}\right)$, which is assumed to known observations, and replace the $x_{a}$ part as the unknown part $Y_{\cdot m}$, which is to be estimated because that the data are missing. Then, we have the expectation of the estimation as

(20) $$E\left[Y_{\cdot m}|\left({\left(Y_{\cdot k}\right)}_{k\in \bar{ℳ}}\right)\right]=\alpha_{m}+\Sigma_{m,\bar{ℳ}}\Sigma_{\bar{ℳ},\bar{ℳ}}^{-1}\left(Y_{\cdot \bar{ℳ}}^{T}-\alpha_{\bar{ℳ}}\right)$$

Then, the estimation of the missing data is calculated as

(21) $${\hat{Y}}_{im}={\hat{\alpha }}_{m}+{\hat{\Sigma }}_{m,\bar{ℳ}}{\hat{\Sigma }}_{\bar{ℳ},\bar{ℳ}}^{-1}\left(Y_{\cdot \bar{ℳ}}^{T}-\alpha_{\bar{ℳ}}\right)$$

Hence, the missing data estimations depend on the estimations of ${\hat{\alpha }}_{m}$, ${\hat{\Sigma }}_{m,\bar{ℳ}}$ and ${\hat{\Sigma }}_{\bar{ℳ},\bar{ℳ}}^{-1}$. Below are assumptions for estimating the model parameters.

**Assumption 1:**  $\forall m\in ℳ,\forall j\in ℐ,\;\left(A_{\cdot m}\;{\left(A_{\cdot j^{\prime }}\right)}_{j^{\prime }\in {ℐ}_{-j}}\right)$*is invertible.*${ℐ}_{-j}=ℐ\backslash \left\{j\right\}$.

**Assumption 2:**  $\forall m\in ℳ,\forall j\in ℐ,Y_{\cdot j}⊥\Omega_{\cdot m}|\left({\left(Y_{\cdot k}\right)}_{k\in \bar{\left\{j\right\}}}\right)$.

Assumption 1 denotes that the matrix $\left(A_{\cdot m}\;{\left(A_{\cdot j^{\prime }}\right)}_{j^{\prime }\in {ℐ}_{-j}}\right)$ is of full rank. **Assumption 2** denotes that, given the values in ${\left(Y_{\cdot k}\right)}_{k\in \bar{\left\{j\right\}}}$, the column $Y_{\cdot j}$ is independent with the column $\Omega_{\cdot m}$.

The missing data imputation for MNAR is to estimate the value of $Y_{im}$ with m∈ℳ for i such that $\Omega_{im}=0$. Assumption 2 leads to

(22) $$E\left[Y_{\cdot j}|\left({\left(Y_{\cdot k}\right)}_{k\in \bar{\left\{j\right\}}}\right)\right]=E\left[Y_{\cdot j}|\left({\left(Y_{\cdot k}\right)}_{k\in \bar{\left\{j\right\}}}\right),\;\Omega_{im}=1\right]$$

### 3.4. Estimation of α

We first define the regression coefficients of $Y_{\cdot j}$ on $Y_{\cdot m}$ and $Y_{\cdot k}$, for $k\in {ℐ}_{-j}$ in the complete case, that will be used to express the mean of a variable with MNAR values.

Considering the model Y=1α+XA+ϵ, with an assumption that matrix $A\in {\mathbb{R}}^{r\times p}$ is of full rank r. Therefore, the expression of $Y_{\cdot j}$ can be reduced to the following linear system.

(23) $$\left(Y_{\cdot m}\;{\left(Y_{\cdot j^{\prime }}\right)}_{j^{\prime }\in {ℐ}_{-j}}\right)=1\alpha_{|r}+\left(X_{\cdot 1},\cdots ,X_{\cdot r}\right)A_{|r}+\epsilon_{|r}$$

Here, $\alpha_{|r}$ and $\epsilon_{|r}$ are the reduced matrix of α and ϵ. $A_{|r}\in {\mathbb{R}}^{r\times r}$ denotes the reduced matrix of $\left(A_{\cdot m}\;{\left(A_{\cdot j^{\prime }}\right)}_{j^{\prime }\in {ℐ}_{-j}}\right)$.

Given Assumption 1, the $A_{|r}$ is invertible, and the inverted matrix is denoted as ${\overset{´}{A}}^{-1}$. The latent matrix of full rank r can be written as

(24) $$\left(X_{\cdot 1},\cdots ,X_{\cdot r}\right)=\left(\left(Y_{\cdot m}\;{\left(Y_{\cdot j^{\prime }}\right)}_{j^{\prime }\in {ℐ}_{-j}}\right)-1\alpha_{|r}-\epsilon_{|r}\right){\overset{´}{A}}^{-1}.$$

Using the original model Y=1α+XA+ϵ, the expression of $Y_{\cdot j}$ is then can be written as

(25) $$Y_{\cdot j}=1\alpha_{j}+\left(\left(Y_{\cdot m}\;{\left(Y_{\cdot j^{\prime }}\right)}_{j^{\prime }\in {ℐ}_{-j}}\right)-1\alpha_{|r}-\epsilon_{|r}\right){\overset{´}{A}}^{-1}A_{j\cdot }+\epsilon_{\cdot j}=\sum_{k\in \left\{m\right\}\cup {ℐ}_{-j}}\left(\sum_{l\in m\cup {ℐ}_{-j}}{\overset{´}{A}}^{-1}{}_{lk}A_{jl}\right)Y_{\cdot k}-\sum_{k\in \left\{m\right\}\cup {ℐ}_{-j}}\left(\sum_{l\in m\cup {ℐ}_{-j}}{\overset{´}{A}}^{-1}{}_{lk}A_{jl}\right)\left(1\alpha_{k}+\epsilon_{\cdot k}\right)+1\alpha_{j}+\epsilon_{\cdot j}\;$$

where we can get the intercept and the coefficients of $Y_{\cdot j}$ on $\left(Y_{\cdot m},{\left(Y_{\cdot k}\right)}_{k\in {ℐ}_{-j}}\right)$.

For $j\in ℐ,\;k\in {ℐ}_{-j}$, let $A_{j\to m,{ℐ}_{-j}\left[0\right]}^{c}$ be the intercept, and $A_{j\to m,{ℐ}_{-j}\left[m\right]}^{c}$ $A_{j\to m,{ℐ}_{-j}\left[k\right]}^{c}$ be the coefficients standing for the effects of $Y_{\cdot j}$ on $\left(Y_{\cdot m},{\left(Y_{\cdot k}\right)}_{k\in {ℐ}_{-j}}\right)$ in the complete case, i.e., when $\Omega_{\cdot m}=1$. Then, we have

(26) $${\left(Y_{\cdot j}\right)}_{|\Omega_{\cdot m}=1}=A_{j\to m,{ℐ}_{-j}\left[0\right]}^{c}+\sum_{j^{\prime }\in {ℐ}_{-j}}A_{j\to m,{ℐ}_{-j}\left[j^{\prime }\right]}^{c}Y_{\cdot j^{\prime }}+A_{j\to m,{ℐ}_{-j}\left[m\right]}^{c}Y_{\cdot k}+\zeta^{c}$$

where the coefficients are calculated as below equations.

(27) $$A_{j\to m,{ℐ}_{-j}\left[j^{\prime }\right]}^{c}=\sum_{l\in \left\{m\right\}\cup {ℐ}_{-j}}{\overset{´}{A}}^{-1}{}_{lj^{\prime }}A_{jl},\;j^{\prime }\in {ℐ}_{-j}$$

(28) $$A_{j\to m,{ℐ}_{-j}\left[m\right]}^{c}=\sum_{l\in \left\{m\right\}\cup {ℐ}_{-j}}{\overset{´}{A}}^{-1}{}_{lm}A_{jl}$$

(29) $$A_{j\to m,{ℐ}_{-j}\left[0\right]}^{c}=1\alpha_{j}-\sum_{k\in \left\{m\right\}\cup {ℐ}_{-j}}\left(\sum_{l\in \left\{m\right\}\cup {ℐ}_{-j}}{\overset{´}{A}}^{-1}{}_{lk}A_{jl}\right)1\alpha_{k}$$

(30) $$\zeta^{c}=-\sum_{k\in \left\{m\right\}\cup {ℐ}_{-j}}A_{j\to m,{ℐ}_{-j}\left[k\right]}^{c}\epsilon_{\cdot k}+\epsilon_{\cdot j}$$

Here, the arrow $j\to m,{ℐ}_{-j}$ indicates the regression model of $Y_{\cdot j}$ on $Y_{\cdot \left(m,{ℐ}_{-j}\right)}$, and the squared bracket [k] indicates the coefficient. Based on the model setting, we have $E\left[\epsilon_{\cdot k}\right]=0$, hence $E\left[\zeta^{c}\right]=0$.

Assumption 2 leads to

(31) $$E\left[Y_{\cdot j}|\left({\left(Y_{\cdot k}\right)}_{k\in \bar{\left\{j\right\}}}\right)\right]=E\left[Y_{\cdot j}|\left({\left(Y_{\cdot k}\right)}_{k\in \bar{\left\{j\right\}}}\right),\;\Omega_{im}=1\right]\;=E\left[A_{j\to m,{ℐ}_{-j}\left[0\right]}^{c}+\sum_{k\in \left\{m\right\}\cup {ℐ}_{-j}}A_{j\to m,{ℐ}_{-j}\left[k\right]}^{c}Y_{\cdot k}+\zeta^{c}|\left({\left(Y_{\cdot k}\right)}_{k\in \bar{\left\{j\right\}}}\right)\right]\;=A_{j\to m,{ℐ}_{-j}\left[0\right]}^{c}+\sum_{k\in \left\{m\right\}\cup {ℐ}_{-j}}A_{j\to m,{ℐ}_{-j}\left[k\right]}^{c}Y_{\cdot k}+E\left[\zeta^{c}|\left({\left(Y_{\cdot k}\right)}_{k\in \bar{\left\{j\right\}}}\right)\right]$$

By taking the expectation of the left and right parts of the equality above given $E\left[\epsilon_{\cdot k}\right]=0$ for $\forall k\in \left\{m\right\}\cup {ℐ}_{-j}$, we have

(32) $$Left=E\left[E\left[Y_{\cdot j}|\left({\left(Y_{\cdot k}\right)}_{k\in \bar{\left\{j\right\}}}\right)\right]\right]=E\left[Y_{\cdot j}\right]=\alpha_{j}$$

(33) $$Right=E\left[A_{j\to m,{ℐ}_{-j}\left[0\right]}^{c}+\sum_{k\in \left\{m\right\}\cup {ℐ}_{-j}}A_{j\to m,{ℐ}_{-j}\left[k\right]}^{c}Y_{\cdot k}+E\left[\zeta^{c}|\left({\left(Y_{\cdot k}\right)}_{k\in \bar{\left\{j\right\}}}\right)\right]\right]\;=A_{j\to m,{ℐ}_{-j}\left[0\right]}^{c}+\sum_{j^{\prime }\in {ℐ}_{-j}}A_{j\to m,{ℐ}_{-j}\left[j^{\prime }\right]}^{c}\alpha_{k}+A_{j\to m,{ℐ}_{-j}\left[m\right]}^{c}\alpha_{m}+E\left[\zeta^{c}\right]$$

Above two equalities are identical. So, we have

(34) $${\hat{\alpha }}_{m}=\frac{\alpha_{j}-A_{j\to m,{ℐ}_{-j}\left[0\right]}^{c}-\sum_{j^{\prime }\in {ℐ}_{-j}}A_{j\to m,{ℐ}_{-j}\left[j^{\prime }\right]}^{c}\alpha_{k}}{A_{j\to m,{ℐ}_{-j}\left[m\right]}^{c}}$$

### 3.5. Estimation of Variance and Covariance

Let $Z=\left(Y_{\cdot k}\right){\;}_{k\in \bar{\left\{\;j\;\right\}}}$, for the variance $\Sigma_{\bar{ℳ},\bar{ℳ}}$, we have

(35) $$\Sigma_{\bar{ℳ},\bar{ℳ}}=Var\left(Y_{\cdot j}\right)=E\left[Var\left(Y_{\cdot j}|Z\right)\right]+Var(E[Y_{\cdot j}|Z]).$$

Assumption 2 leads to $Var\left(Y_{\cdot j}|Z\right)=Var\left(Y_{\cdot j}|Z,\Omega_{\cdot m}=1\right)$. According to the conditional variance for a Gaussian vector, we have

(36) $$Var\left(Y_{\cdot j}|Z\right)=Var\left(Y_{\cdot j}\right)-Cov\left(Z,Y_{\cdot j}\right)Var{\left(Z\right)}^{-1}Cov{\left(Z,Y_{\cdot j}\right)}^{T}.$$

Then, we have the first term of $Var\left(Y_{\cdot j}\right)$ as

(37) $$E\left[Var\left(Y_{\cdot j}|Z\right)\right]=Var\left(Y_{\cdot j}\right)-Cov\left(Z,Y_{\cdot j}\right)Var{\left(Z\right)}^{-1}Cov{\left(Z,Y_{\cdot j}\right)}^{T}|\Omega_{\cdot m}=1$$

For the second term of $Var\left(Y_{\cdot j}\right)$, we have

(38) $$Var\left(E\left[Y_{\cdot j}|Z\right]\right)=Var\left(E\left[Y_{\cdot j}|Z,\Omega_{\cdot m}=1\right]\right)\;=Var\left(\sum_{k\in \left\{m\right\}\cup {ℐ}_{-j}}A_{j\to m,{ℐ}_{-j}\left[k\right]}^{c}Y_{\cdot k}-\sum_{k\in \left\{m\right\}\cup {ℐ}_{-j}}A_{j\to m,{ℐ}_{-j}\left[k\right]}^{c}E\left[\epsilon_{\cdot k}|Z\right]+A_{j\to m,{ℐ}_{-j}\left[0\right]}^{c}+E\left[\epsilon_{\cdot j}\right]\right)$$

where $E\left[\epsilon_{\cdot k}|Z\right]=\sigma^{2}{\left(Var{\left(Z\right)}^{-1}\right)}_{k\cdot }\left(Z-E\left[Z\right]\right)$.

For the covariances ${\hat{\Sigma }}_{m,\bar{ℳ}}$ between $Y_{\cdot m},$ $Y_{\cdot k}$, k∈ℐ, let $Z=\left(Y_{\cdot l}\right){\;}_{l\in \bar{\left\{\;k\;\right\}}}$, we have

(39) $${\hat{\Sigma }}_{m,\bar{ℳ}}=Cov\left(Y_{\cdot m}\;Y_{\cdot k}\right)=E\left[Y_{\cdot m}\;Y_{\cdot k}\right]-E\left[Y_{\cdot m}\right]E\left[\;Y_{\cdot k}\right]\;=E[E[Y_{\cdot m}Y_{\cdot k}|Z]]-E\left[Y_{\cdot m}\right]E\left[\;Y_{\cdot k}\right]\;=E\left[Y_{\cdot m}E\left[Y_{\cdot k}|Z\right]\right]-E\left[Y_{\cdot m}\right]E\left[\;Y_{\cdot k}\right].\;$$

For the first term, we have

(40) $$E\left[Y_{\cdot m}E\left[Y_{\cdot k}|Z\right]\right]=E\left[Y_{\cdot m}E\left[Y_{\cdot k}|Z,\Omega_{\cdot m}=1\right]\right]\;=E\left[Y_{\cdot m}\left(A_{j\to m,{ℐ}_{-j}\left[0\right]}^{c}+\underset{j^{\prime }\in \left\{m\right\}{ℐ}_{-j}}{\sum }A_{j\to m,{ℐ}_{-j}\left[j^{\prime }\right]}^{c}Y_{\cdot j^{\prime }}+E\left(\zeta^{c}|Z\right)\right)\right]\;=A_{j\to m,{ℐ}_{-j}\left[0\right]}^{c}E\left[Y_{\cdot m}\right]+A_{j\to m,{ℐ}_{-j}\left[m\right]}^{c}E\left[Y_{\cdot m}^{2}\right]+\underset{j^{\prime }\in {ℐ}_{-j}}{\sum }A_{j\to m,{ℐ}_{-j}\left[j^{\prime }\right]}^{c}E\left[Y_{\cdot m}Y_{\cdot j^{\prime }}\right]+E\left[Y_{\cdot m}E\left(\zeta^{c}|Z\right)\right]$$

According to the derivation in [22], $E\left[Y_{\cdot m}E\left(\zeta^{c}|Z\right)\right]$ is calculated as

(41) $$-\sigma^{2}\left(\sum_{l\in {ℐ}_{-k}}\sum_{s\in {ℐ}_{-k}}Var{\left(Z\right)}^{-1}A_{j\to m,{ℐ}_{-j}\left[l\right]}^{c}Cov\left(Y_{\cdot m}Y_{\cdot l}\right)+A_{j\to m,{ℐ}_{-j}\left[m\right]}^{c}\right).$$

Note that for the second term, $E\left[Y_{\cdot m}\right]E\left[\;Y_{\cdot k}\right]$, it can be directly calculated.

## 4. Experiment

### 4.1. Dataset and Preprocessing

We utilize a road traffic speed dataset published by [23]. Road segments are anonymous, covering the main urban expressways within two months from 1 August to 30 September 2016, (a total of 61 days).

The time interval is 10 min. From the original dataset, we select twenty links whose speed are generated in the morning rush hours (i.e., 7:00 A.M. to 9:00 A.M.) for evaluating the proposed method. The speed of each link is transformed to the congestion index, which is calculated as vijmax(v·j), $v_{ij}$ denotes the $i^{}$ speed value of link j and $v_{\cdot j}$ denotes all observations of link j. For each link j, the time series length of speed observations is 732 (12 observations in two hours 61 days). Hence, the dimension of Y is n=732, p is the number of links.

**Figure 1:**
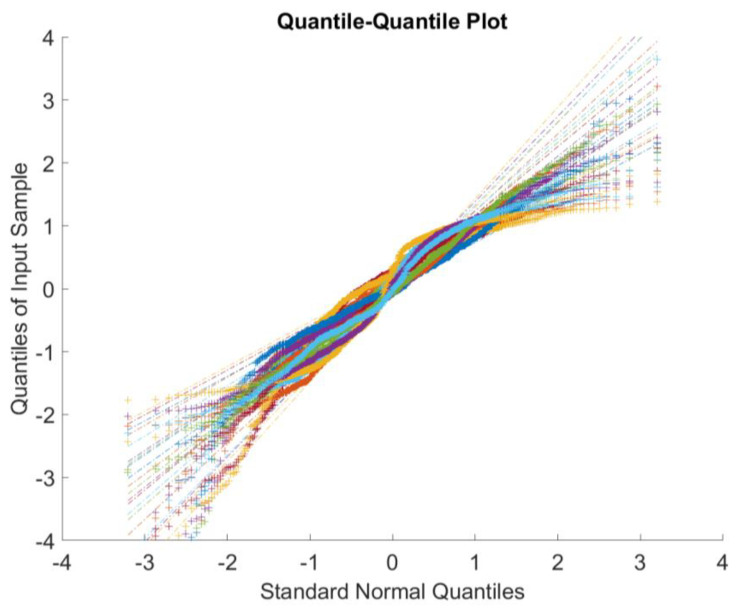
Plot of the Data Quantitles and Standard Normal Quantitles.

The basic assumption of the proposed model is that the observations of each link are drawn from a Gaussian distribution, Hence, we adopt the quantile–quantile plot (QQ Plot) to display the quantiles of the data (after normalizing) versus the theoretical quantile values from a normal distribution. If the distribution of the data is normal, then the data plot is linear. As shown in the Figure 1, the plot closely follows the straight lines, suggesting that the data after normalizing the congestion data have an approximately normal distribution.

### 4.2. Metrics for Missing Data Imputation Accuracy

For evaluating the performance of missing data imputation, we adopt the below four metrics, including Root Mean Square Error (RMSE), Mean Absolute Error (MAE), Symmetric Mean Absolute Percentage Error (SMAPE), and R^2^. Note that a higher R^2^ value denotes better accuracy.

$$\mathrm{RMSE}=\sqrt{\frac{1}{n}\sum_{i=1}^{n}{\left(y_{i}-{\hat{y}}_{i}\right)}^{2}}\;\mathrm{MA}E=\frac{1}{n}\sum_{i=1}^{n}\left|y_{i}-{\hat{y}}_{i}\right|\;\mathrm{SMA}\mathrm{PE}=\frac{1}{n}\sum_{i=1}^{n}\frac{\left|y_{i}-{\hat{y}}_{i}\right|}{\left(|y_{i}|+\left|{\hat{y}}_{i}\right|\right)/2}\;R^{2}=1-\frac{\sum_{i=1}^{n}{\left(y_{i}-{\hat{y}}_{i}\right)}^{2}}{\sum_{i=1}^{n}{\left(y_{i}-{\bar{y}}_{i}\right)}^{2}}$$

### 4.3. Benchmark and Experiment Settings

We compare the new model with the typically used PPCA model, where $\sigma^{2}$ and A is estimated by an expectation-maximum (EM) algorithm. We name it ppca-em in this section. We further use the estimated $\sigma^{2}$ by EM as the known inputs of the new model in this study. As to the rank r in the model, the best value is determined by cross-validation on the dataset. In this section, we further detail the experiment settings in terms of the MNAR data generation and the settings of link set ℳ.

#### 4.3.1. Generating MNAR

Note that the model targets at solving the imputation for MNAR data. We utilize the mechanism of generating MNAR in [22]. Specifically, a logistic regression function is adopted as f(x)=1/(−a(x−b)), where x is an observation, and (a,b) is set for selecting different missing percentage. The function transforms the observation x to a value in (0,1). The observation x with f(x)>μ, is set to be the MNAR data, where μ is a random threshold. We set the parameters (a,b) as below Table 1, which is corresponding to a specific missing percentage.

#### 4.3.2. Settings of Link Set ℳ

Missing data on different links may obtain different recover accuracy, even with the same missing percentage. For evaluating this proposition, we first test the missing data imputation accuracy with different p-score values of the links. When a link observation $Y_{\cdot j}$ is set to be ℳ={j}, all other links are set to be ${ℐ}_{-j}=ℐ\backslash \left\{j\right\}$, where ℐ={1,2,…,20}. Further, we test the missing data imputation accuracy of several select links (or link combination) compared with the ppca-em model, to evaluate the advantage of the new model.

### 4.4. Results and Analysis

**Figure 2:**
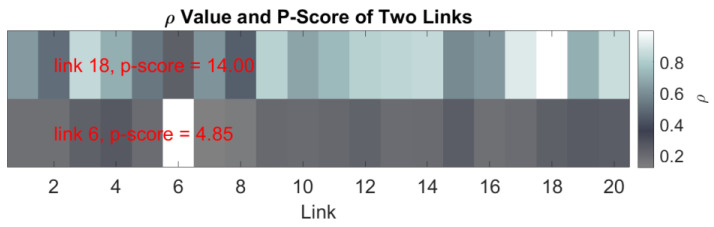
*ρ* Value of Two Links with the Highest and Lowest p-score.

We first examine the relationship between the missing data imputation accuracy and the proposed metric, i.e., p-score value. We select two links with the highest p-score value and the lowest p-score value in the dataset. The p-score values of two selected links, i.e., link 6 and link 18, are shown in Figure 2, where the color map denotes the ρ values between the selected link and all links in link set ℐ.

**Figure 3:**
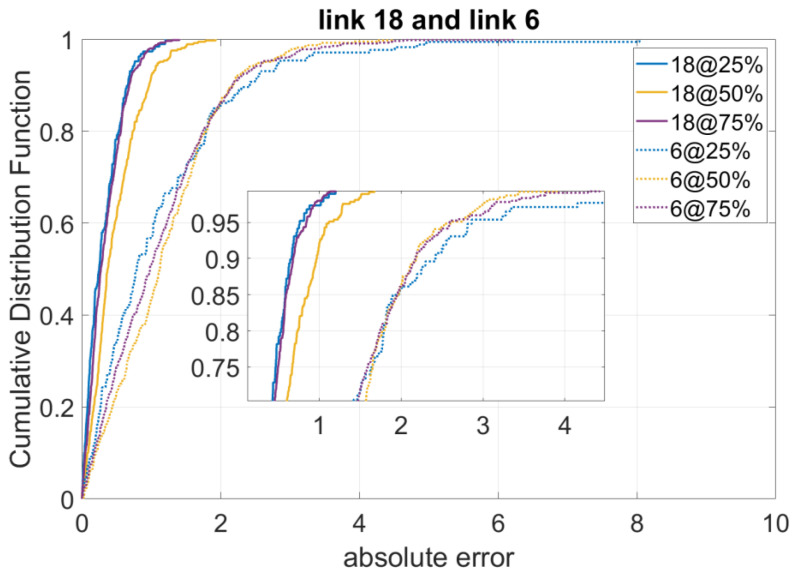
Performance of the Algorithm for Links with Highest p-Score and Lowest p-Score.

Accordingly, we calculate the absolute errors of the model on these two selected links. Figure 3 shows the results missing data imputation results on these links in terms of different missing data percentages. We can see that missing data on link 18, which is with a higher p-score value than that of link 6, are better recovered regarding all scenarios of missing data percentages (25%, 50%, 75%).

**Figure 4:**
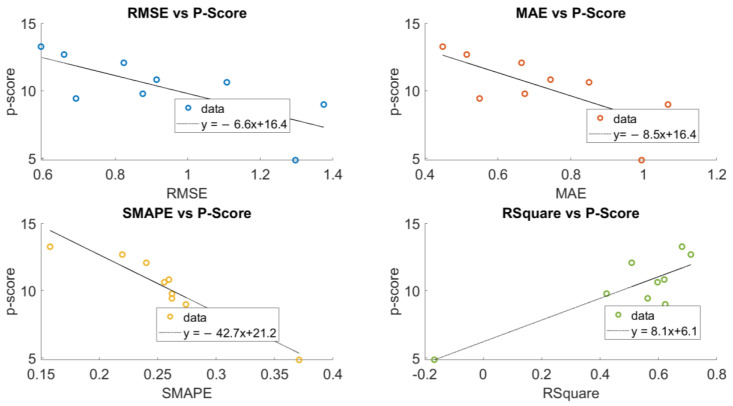
Scatter Plot between the Accuracy Metrics and the p-score Values.

We further examine the relationship between the p-score value and the accuracy metrics on the traffic dataset, which is shown in below Figure 4. The accuracy measured by four metrics presents a positive correlation with the p-score value on different links, meaning that missing data on the links with higher p-score values can be better recovered.

We also compare the new model with the ppca-em model on other links or link combinations in the dataset. The settings and corresponding missing data imputation results measured by the four metrics are shown in Table 2. Except the above four metrics, we further added the accuracy as another metric for directly representing the estimation accuracy results and better understanding the accuracy comparison between the new model and the baseline. Here, the accuracy is calculated as

$$\mathrm{Accuracy}=1-100\%*\frac{1}{n}\sum_{i=1}^{n}\frac{\left|y_{i}-{\hat{y}}_{i}\right|}{|y_{i}|+\left|{\hat{y}}_{i}\right|}$$

**Figure 5:**
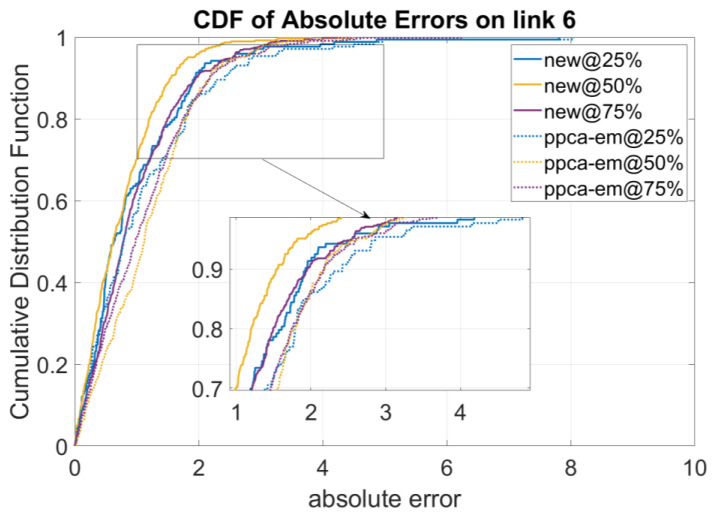
Performance of Models on the Link with Lowest p-Score Values.

Note that Figure 3 already shows that the new model obtains the worst accuracy on link 6. Hence, we further compare two models on this link to compare the new model with the ppca-em model. The results are shown in Figure 5. It shows that even on link 6, the absolute errors of the new model are still lower than the ppca-em model for three missing ratios.

The experiment results in Table 2 and Figure 5 demonstrate that the new model performs better than the typically used ppca-em model in terms of four accuracy metrics and computing time. The results indicate that the new model is more effective and efficient for the MNAR traffic data imputation problem, which is the target of this study. The typical ppca-em method is usually used for imputation of data missing at random, whereas the new model is more general and is capable of MNAR data imputation.

## 5. Discussion

Our improved linear probabilistic principal component analysis method can be applied to a variety of missing traffic data imputation applications, such as missing traffic speed estimation, or other traffic indicators. Notably, because the proposed missing data imputation method is a linear and interpretable model, which is naturally of high computing efficiency, it can be utilized in the systems where real-time missing data estimation is required. Additionally, the time-series based metric, p-Score value, is proposed to distinguish variables, e.g., links with missing traffic speed data, for estimating the missing values. Such a method can be applied to the applications of traffic surveillance systems to identify which sensors should be of high priority to maintained in the systems to ensure the full surveillance, or which links should be equipped with sensor for traffic surveillance.

## 6. Conclusions

In this study, we propose a general linear model based on the PPCA to tackle the MNAR traffic data imputation problem. We also propose a time series-based metric, i.e., the p-score, to distinguish links that are of missing data. Experimental results on a real-world traffic dataset show that the proposed model performs better than the typically used ppca-em model in terms of missing data imputation accuracy and computing time. Furthermore, we test the model on links with different p-score values. The experiment results show that the missing data on links with higher p-score values are better recovered. Such an observation helps us understand the data recovering distinction for different links in the road network, which has not been studied in any research to our best knowledge. In future work, we will further compare the model with other methods on more traffic datasets.

## Figures and Tables

**Table 1:** Settings for Generating MNAR Data in the Experiments.

a	b	Missing Percentage
−1	−1.3	25%
3	0	50%
1	−1.3	75%

**Table 2:** Experiment Setting and performance of the algorithms with different Percent of MNAR Data on Links.

**Experiment Setting: Missing Rate (%)** @ ℳ										
	50 @{1}		50 @{3}		75 @{1}		75 @{1,3}		75 @{3,5}	
p-score	10.62@{1}		13.26@{3}		10.62@{1}		−		9.42@{5}	
**Per** **formance Comparison**										
Metrics	ppca-em	New	ppca-em	New	ppca-em	New	ppca-em	New	ppca-em	New
RMSE	0.992	0.746	0.559	0.595	1.069	0.746	0.835	0.871	0.942	0.627
MAE	0.810	0.564	0.458	0.448	0.789	0.564	0.598	0.625	0.665	0.468
SMAPE	0.340	0.223	0.216	0.157	0.289	0.223	0.231	0.228	0.253	0.201
R^2^	0.150	0.688	0.595	0.681	0.545	0.688	0.208	0.677	0.115	0.740
Accuracy	83.0%	88.9%	89.2%	92.2%	85.5%	88.9%	88.4%	88.6%	87.3%	89.9%
**Computing Time**										
Sec	6.54	2.03	6.29	2.03	6.73	2.64	6.06	4.06	11.32	4.11

## Data Availability

The dataset used in this paper is published by [23], which can be found at https://doi.org/10.5281/zenodo.1205229, Access on 18 February 2022.
